# Multiple sclerosis patients under treatment with interferon β1-a or ocrelizumab exhibit different T and B cell responses to SARS-CoV-2 vaccine

**DOI:** 10.3389/fimmu.2026.1773417

**Published:** 2026-06-16

**Authors:** Antonietta Liotti, Giorgia Teresa Maniscalco, Anne Lise Ferrara, Valeria Mazzone, Maedeh Dehbozorgi, Annamária Szabó, Martina Belardo, Daria Marcogiuseppe, Antonio De Martino, Maria Elena Di Battista, Giovanna Servillo, Ornella Moreggia, Daniele Di Giulio Cesare, Vincenzo Andreone, Gilda Varricchi, Veronica De Rosa

**Affiliations:** 1 Centro Interdipartimentale di ricerca in Scienze Immunologiche di Base e Cliniche (CISI), University of Naples Federico II, World Allergy Organization (WAO) Center of Excellence, (CoE), Naples, Italy; 2 Centro di Neuroimmunologia e Sclerosi Multipla, Ospedale “A.Cardarelli”, Naples, Italy; 3 Istituto degli Endotipi in Oncologia, Metabolismo e Immunologia “G. Salvatore”, Consiglio Nazionale delle Ricerche (CNR), Naples, Italy; 4 Dipartimento di Scienze Mediche Cliniche e Sperimentali, Università della Campania “Luigi Vanvitelli”, Naples, Italy; 5 Dipartimento di Medicina Molecolare e Biotecnologie Mediche, University of Naples Federico II, Naples, Italy; 6 Dipartimento di Scienze Mediche Traslazionali, University of Naples Federico II, Naples, Italy

**Keywords:** disease-modifying therapies, interferon beta1-a, multiple sclerosis, ocrelizumab, SARS-CoV-2 vaccine, T/B cell response, Tregs

## Abstract

**Introduction:**

Multiple sclerosis (MS) is a long-term autoimmune disease characterized by inflammation and progressive degeneration of the central nervous system. Disease-modifying therapies (DMTs) have proved to be effective at ameliorating the course of MS. DMTs are immunomodulatory drugs affecting immune response to SARS-CoV-2 and its vaccine efficacy in MS patients (pwMS).

**Methods:**

In this longitudinal study, we analyzed T and B cell subsets in peripheral blood and the antigen (spike)-specific T cell response in pwMS patients undergoing interferon β1-a (IFN) or ocrelizumab (OCRE), before and after BNT162b2 mRNA SARS-CoV-2 vaccination. Blood was collected from pwMS treated with IFN and OCRE before and after the two vaccine doses. Peripheral blood mononuclear cells were analyzed by flow cytometry to measure T and B cell frequency, the expression of activation/regulatory/memory T cell markers and their spike-specific T cell response.

**Results:**

CD20^+^ B-cells were decreased in OCRE- compared to IFN- treated pwMS before and after vaccination. CD8^+^ and CD4^+^ T cell responses and the immunological memory was comparable between the two groups. Th17 cells were increased after vaccination only in the OCRE-treated group. We detected a robust spike-specific T cell response, paralleled by a significant decrease of Treg frequency, in both pwMS groups.

**Discussion:**

Although IFN and OCRE induced distinct immunological profile, both DMTs allowed to mount a vaccine-induced cellular and humoral immune response. This study also represents a model that could be applied to dissect the vaccine-elicited immune response in individuals undergoing treatment with different biological drugs.

## 1 Introduction

Multiple sclerosis (MS) is a chronic inflammatory and neurodegenerative disorder of the central nervous system and spinal cord, affecting over 2.5 million people worldwide (1). The pathogenesis of MS is complex, involving genetic, environmental and immunological factors (2, 3). Traditionally, MS has been considered an autoimmune disease, with autoreactive T lymphocytes attacking myelin sheaths and B cells contributing to chronic low-grade inflammation (1). A range of disease-modifying therapies (DMTs) have proved to be effective at ameliorating the course of MS (4). DMTs modulate and/or suppress specific aspects of the immune system, potentially increasing susceptibility to infections and reducing vaccine efficacy (5). Specifically, DMTs may impair vaccine-induced immune response against different types of viral infections, such as severe acute respiratory syndrome coronavirus-2 (SARS-CoV-2) (6–8). Hence, it is of paramount importance to determine the immunosuppressive effects of specific DMT in patients with MS (pwMS) during vaccination.

SARS-CoV-2 vaccination induces protective immunity through a combination of both humoral and cellular responses by orchestrating a complex functional network of B and T lymphocytes [CD8^+^, CD4^+^ and regulatory T cells (Tregs)] (9). The efficacy of SARS-CoV-2 mRNA vaccination in inducing cellular and humoral immune responses in immunocompetent subjects has been well established (10–12). In contrast, the cellular and humoral responses to SARS-CoV-2 vaccination in pwMS treated with different DMTs have been rather controversial. Several studies reported a reduced humoral and/or cellular immune responses after SARS-CoV-2 vaccination in pwMS during therapy with a humanized anti-CD20 monoclonal antibody (mAb) (ocrelizumab: OCRE) (13–16) or sphingosine-1-phosphate receptor (S1PR) modulators (14, 17–21). By contrast, other authors have demonstrated robust antigen-specific cellular and humoral immune responses after SARS-CoV-2 vaccination in pwMS treated with anti-CD20 (13, 22–24) or interferon β1-a (25).

Although OCRE depletes/impairs B cells and T cell subsets (26–28), it has been suggested that these effects might contribute to its therapeutic effects in pwMS (29, 30). Interferon (IFN)β1-a, the recombinant form of the naturally occurring cytokine IFN, increases anti-inflammatory cytokines, reduces leukocyte trafficking across the blood-brain barrier (BBB) and inhibits T cell activation (12). IFN is one of the first FDA-approved drugs and is widely used as DMT first-line treatment in MS (13). OCRE induces B cell depletion and was approved by the FDA in 2017 for the treatment of pwMS (31).

Recently, novel SARS-CoV-2 variants were reported (32), recording new peaks of infection and raising attention on the potential of COVID-19 disease in fragile subjects with comorbidities (8), such as pwMS. In this scenario, a detailed evaluation of the vaccine immune response in pwMS is needed both for the comprehension of its effectiveness and enabling a rapid intervention in this high-risk group, should another pandemic occur.

In this longitudinal study, we analyzed peripheral blood T and B cell subsets to assess the immunological phenotype in pwMS under IFN or OCRE treatment, before, during and after two doses of BNT162b2 mRNA SARS-CoV-2 vaccination. We also evaluated the antigen (spike)-specific T cell response in CD8^+^ and CD4^+^ T cells isolated from the same cohort of pwMS.

## 2 Materials and methods

### 2.1 Subjects and study design

This was a longitudinal monocentric study aiming at evaluating the immunological phenotype and T and B cell responses after BNT162b2 vaccine in pwMS undergoing two different DMTs therapies. Subjects with MS were enrolled at the Neuroimmunology and Multiple Sclerosis Center of the Cardarelli Hospital (Naples, Italy) from March 2021 to June 2021. A total of 20 pwMS were enrolled after obtaining informed consent. Ten patients were treated with IFN β1-a and 10 pwMS were treated with OCRE. None of the enrolled patients had previously been affected by SARS-CoV-2. pwMS treated with IFN β1-a were vaccinated without any interruption of immunomodulatory treatment; OCRE-treated pwMS were vaccinated at least 1 to 3 months after the last administration, according to the recommendations of Italian Authority of Health (33). Demographic and clinical characteristics of the study cohort are shown in **Table 1**. Inclusion criteria were: patients aged between 18 and 65 years, diagnosed with MS and treated with DMTs for at least 6 months. Exclusion criteria were: previous SARS-CoV-2 infection, any relapse and/or glucocorticoid treatment in the last 30 days before enrolment and pregnancy.

**Table 1:** Demographic and clinical characteristics of the study cohort.

Cohort description	IFN-group (n=10)	OCRE-group (n=10)	*p*-value
Gender, n (%)			0.07
Male	2 (20)	7 (70)	
Female	8 (80)	3 (30)	
Age, years			0.79
Mean age (± SD)	40.1 (± 7.9)	39.6 (± 14.2)	
MS type, n (%)			0.08
RRMS	10 (100)	6 (60)	
PPMS	0 (0)	1 (10)	
SPMS	0 (0)	3 (30)	
EDSS, mean (range)	1 (0 - 2.5)	4 (1 – 6.5)	**0.02**
Disease duration, mean (± SD), months	116.9 (± 58.3)	107 (± 97.4)	0.52
DMTs duration, mean (± SD), months	106.4 (± 50)	17 (± 10.7)	**0.001**

DMTs, Disease modifying therapy; EDSS, Expanded disability status scale; IFN, Interferon β1-a; IQR, Interquartile range; MS, Multiple sclerosis; OCRE, Ocrelizumab; PPMS, Primary progressive multiple sclerosis; RRMS, Relapsing remitting multiple sclerosis; SPMS, Secondary progressive multiple sclerosis.Bold values refer to tatistically significant differences.

### 2.2 Blood collection and isolation of mononuclear cells

Whole blood was collected into heparinized Vacutainers^®^ (BD Biosciences) and processed within the following 4 hours. Peripheral blood mononuclear cells (PBMCs) were isolated by density-gradient sedimentation using Ficoll-Paque (Lymphoprep, Nycomed Pharma, Oslo, Norway) according to standard procedures (34, 35). PBMCs were cryopreserved in media containing 10% DMSO (Sigma-Aldrich) and 90% foetal bovine serum (FBS) (GIBCO) and stored until used.

### 2.3 Flow cytometry analysis of blood-derived B and T lymphocytes

Freshly isolated PBMCs from IFN- and OCRE-treated pwMS were surface-stained for 20 minutes at 4 °C with the following mAbs: FITC-conjugated anti-human CD19 (BD Pharmigen, clone: SJ25C1), Pacific Blue-conjugated anti-human CD8 (BD Horizon, clone: RPA-T8), APC-Cy7-conjugated anti-human CD4 (BD Pharmigen, clone: RPA-T4), PerCP Cy5.5-conjugated anti-human CD69 (BD Bioscience, clone: L78), PE-Cy7-conjugated anti-human TIGIT (Thermofisher, clone: MBSA-43), APC-conjugated anti-human OX40 (BD Pharmigen, clone: ACT35), AmCyan-conjugated anti-human CD62L (BD Horizon, clone: DREG56) and BV421–conjugated anti-human CD279/PD-1 (BD Biosciences, clone: EH12.1). Thereafter, cells were washed, fixed and permeabilized (anti-human FOXP3 staining Set PE; eBioscience) and intracellular stained with following mAbs: PE-conjugated anti-human CD154 (BD Pharmigen, clone: TRAP1), BV421-conjugated anti-human RORγT (BD Horizon, clone: Q21-559); PE-conjugated anti-human FOXP3 (eBioscience, clone: PCH101, APC–conjugated anti-human CD152/CTLA-4 (BD Biosciences, clone: BNI3), Alexa Fluor 488–conjugated anti-human Helios (BD Biosciences, clone: 22F6) and BV510–conjugated anti-human Ki67 (BD Biosciences, clone: B56). Cells were analysed with a MACS Quant Analyzer 10 (Miltenyi Biotec, Bergisch Gladbach, Germany) and analyzed with FlowJo software (v.10).

### 2.4 Activation induced cell marker and intracellular cytokine assay

Frozen PBMCs were thawed and diluted in complete RPMI 1640 with 10% human AB serum (Biowest, Nuaillé, France) and rested for at least 4 hours. After that, cells were cultured in 96-well plate in the presence of SARS-CoV-2 peptide pool (1 µg/ml) covering the complete protein coding sequence (amino acids 5–1273) of the surface or spike glycoprotein (“S”) of SARS Coronavirus 2 (GenBank MN908947.3, Protein QHD43416.1) (PepTivator SARS-CoV-2 Prot_S complete, Miltenyi Biotec, Bergisch Gladbach, Germany). PBMCs were stimulated for 18 hours at 37 °C in a 5% CO_2_ atmosphere in complete culture medium (RPMI 1640 supplemented with 5% human serum and 1% of L-glutamine, sodium pyruvate, nonessential amino acids, antibiotics, 0.1M HEPES). For each stimulated sample, an unstimulated one was prepared, as negative control. Golgi-Plug containing brefeldin A (BD Biosciences) (36) was added 3 hours into cell culture. After the stimulation, cells were washed and surface stained for 20 minutes at 4 °C with the following antibodies: BV510-conjugated anti-human CD3 (Miltenyi, clone: BW264/56), APC-conjugated anti-human CD134 (OX40) (BD Pharmigen, clone: ACT35), APC-H7-conjugated anti-human CD4 (BD Pharmigen, clone: RPA-T4), PE-conjugated anti-human CD69 (Miltenyi, clone: FN50), PE-Cy 7-conjugated anti-human CD8 (BD Horizon, clone: RPA-T8). Thereafter, cells were washed, fixed and permeabilized (fixation-permeabilization buffer; eBioscience) and intracellularly stained for 30 minutes at 4 °C with: PE-conjugated anti-human CD154 (BD Pharmigen, clone: TRAP1), BV421-conjugated anti-human IFNγ (BD Horizon, clone: 4S.B3), FITC-conjugated anti-human TNFα (Miltenyi, clone: cA2), PerCP-conjugated anti-human IL-2 (BD Pharmigen, clone: MQ1-17H12). Flow cytometry acquisitions were performed with MACSQuant Analyser 10 (Miltenyi).

### 2.5 Ethics committee

This study was conducted according to the Good Clinical Practice guidelines and the ethical principles of the Declaration of Helsinki. Investigators obtained approval for the study protocol and amendments by the Ethics Committee of A.O.R.N. A. Cardarelli/Santobono-Pausilipon (protocol n. 2821). All subjects gave written informed consent to participate in the study.

### 2.6 Statistical analysis

Descriptive analyses were presented as mean (± standard error of the mean), median and interquartile range (IQR). Categorial variables were described as frequency and percentage. A Shapiro-wilk test was performed to assess the normal distribution of data. In cases of non-normal distribution, appropriate non-parametric tests were applied (Wilcoxon for paired and Mann-Whitney for unpaired data). *p*-value less than 0.05 was considered statistically significant. All statistical analyses and data visualization were performed using Prism 8.0 software (Graphpad Software, San Diego, CA, USA).

## 3 Results

### 3.1 Study population

**Figure 1:**
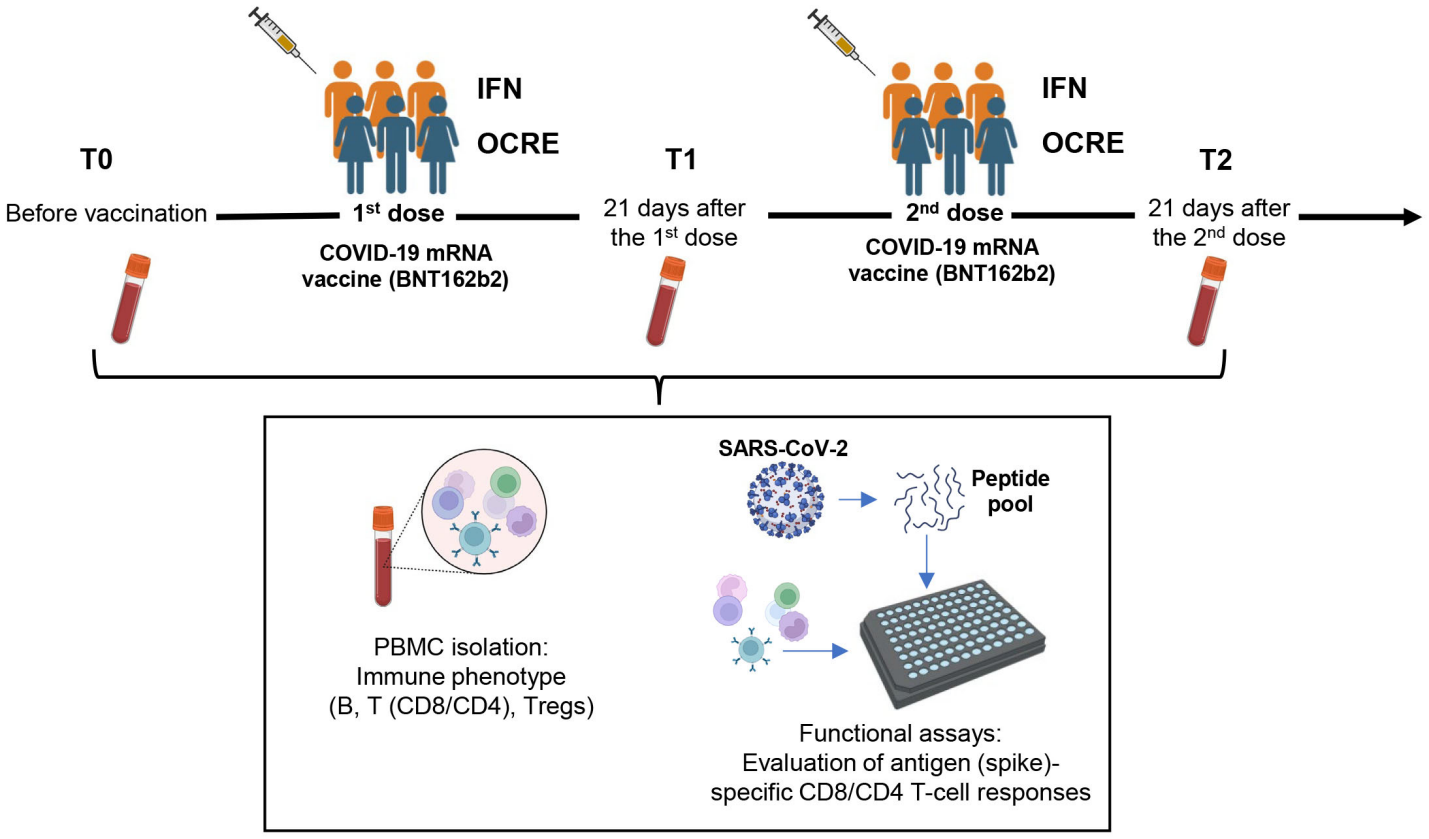
Schematic representation of study design. 20 patients [10 treated with Interferon β1-a (IFN) and 10 treated with ocrelizumab (OCRE)] were enrolled and followed during BNT162b2 mRNA vaccination. Blood samples were obtained from each patient before vaccination (T0), 21 days after the first (T1) and the second (T2) vaccine dose. Peripheral blood mononuclear cells (PMBCs) were isolated and characterized to evaluate T and B lymphocyte subsets by flow cytometry. Activation induced cell markers and antigen (spike)-specific CD8/CD4 T-cell responses were also evaluated. The figure was designed with Biorender.com.

In this longitudinal monocentric study, we enrolled 20 patients with multiple sclerosis (pwMS) undergoing two different disease-modifying therapies (DMTs): Interferon β1-a (IFN; n=10) and ocrelizumab (OCRE; n=10). Patients were followed throughout the entire BNT162b2 vaccination cycle: before the vaccine (T0), 21 days after the first (T1) and 21 days after the second (T2) dose. The vaccination and blood withdrawal schedule are reported in **Figure 1**. The demographic and clinical characteristics of the study cohort are reported in **Table 1**. pwMS in the IFN group were 80% female and 20% male (mean age: 40.1 ± 7.9), while those in the OCRE group were 30% female and 70% male (mean age: 39.6 ± 14.2). IFN-treated pwMS were all relapsing-remitting (RR), while 60% were relapsing remitting multiple sclerosis (RRMS), 10% primary progressive (PP) and 30% (n=3) secondary-progressive (SP) in the OCRE group. MS patients did not experience any relapse or disease progression during the observation period post-vaccination.

### 3.2 Immunological profile of INF- and OCRE-treated pwMS undergoing BNT162b2 vaccination

We performed a whole blood-basic immunological analysis of pwMS and measured the anti-Spike IgG titre as readout of the humoral response (**Tables 2**-**4**). We detected a robust anti-Spike IgG titre after the first and second vaccination doses only in IFN-treated pwMS (**Tables 2**, **3**), as previously reported (25, 37). The low anti-Spike IgG titre in the OCRE-group was associated with a reduction in the absolute number of B cells (CD20^+^) at baseline (T0) and throughout the vaccination (T1 and T2) (**Tables 2**, **4****),** consistent with prior findings (23, 38–40). We also found a slight but significant increase in CD8^+^ T cells in the OCRE-treated pwMS at baseline (T0) compared to the IFN group (**Table 2**).

**Table 2:** Peripheral blood cell counts in the multiple sclerosis (MS) cohort before and during vaccination.

Blood cells	T0, mean (± sd)		*p*-value	T1, mean (± sd)		*p*-value	T2, mean (± sd)		*p*-value
	IFN	OCRE		IFN	OCRE		IFN	OCRE	
WBC (cells/μL)	6137 (± 1574)	6997 (± 1668)	0.16	5943 (± 875)	6249 (± 1969)	0.79	6442 (± 1486)	6874 (± 1456)	0.65
Lymphocytes (cells/μL)	1974 (± 702)	1657 (± 338)	0.47	1881 (± 588)	1781 (± 505)	0.63	1974 (± 667)	1649 (± 299)	0.41
CD20^+^ (cells/µL)	237 (± 118)	15.03 (± 30.7)	**<0.001**	213.9 (± 114)	48.09 (± 82.7)	**0.002**	238.2 (± 145)	7.9 (± 20)	**0.0002**
CD3^+^ (cells/μL)	1455 (± 557)	1378 (± 329)	0.97	1445 (± 475)	1459 (± 361)	0.91	1450 (± 470)	1312 (± 283)	0.54
CD4^+^ (cells/µL)	980 (± 437)	853 (± 256)	0.85	1014 (± 404)	927 (± 298)	0.63	1029 (± 387)	818 (± 214)	0.27
CD8^+^ (cells/µL)	197 (± 50.7)	249 (± 48.3)	**0.04**	218 (± 60.8)	248 (± 87.5)	0.39	205 (± 52.6)	261 (± 105)	0.20
IgG Spike (U/mL)	52.8 (± 166)	0.50 (± 0.26)	0.33	1293 (± 3938)	27 (± 75.6)	**0.01**	3078 (± 3459)	283 (± 537)	**0.0009**

Absolute number of white blood cells (WBC), lymphocytes, CD20^+^, CD3^+^, CD4^+^ and CD8^+^ cells in IFN- *vs* OCRE-treated MS subjects undergoing BNT162b2 vaccination.

IFN, Interferon β1-a; OCRE, Ocrelizumab; SD, standard deviation; WBC, white blood cell.Bold values refer to tatistically significant differences.

**Table 3:** Peripheral blood cell counts in the IFN-treated pwMS cohort during vaccination.

Blood cells	IFN			*p value*
	T0 (mean ± sd)	T1 (mean ± sd)	T2 (mean ± sd)	
WBC (cells/µL)	6137 (± 1573.6)	5943 (± 875.5)	6442 (± 1486.3)	–
Lymphocytes (cells/µL)	1974 (± 702)	1881 (± 587.7)	1974 (± 667)	-
CD20^+^ (cells/µL)	236.9 (± 118.1)	213.9 (± 113.5)	238.2 (± 145.4)	-
CD3^+^ (cells/µL)	1455.4 (± 557)	1445 (± 475)	1450 (± 469.7)	-
CD4^+^ (cells/µL)	980.2 (± 439.6)	1014 (± 403.5)	1029 (± 386.5)	–
CD8^+^ (cells/µL)	197.5 (± 50.7)	217.7(± 60.8)	205.2 (± 52.6)	-
IgG Spike	52.8 (± 165.8)	1293 (± 3938)	3078.5 (± 3459)	**; ##; ¥¥

The absolute number of white blood cells (WBC), lymphocytes, CD20^+^, CD3^+^, CD4^+^, and CD8^+^ in IFN-treated MS subjects undergoing BNT162b2 vaccination was evaluated before the vaccine (T0), 21 days after the first (T1) and the second (T2) vaccine dose.

Data are presented as mean ± sd and statistical analysis was performed using the Wilcoxon signed-rank test (two tails); **;^##^;^¥¥^ p<0.01. * T0 *vs* T1; ^#^ T1 *vs* T2; ^¥^ T0 *vs* T2.

IFN, Interferon β1-a; SD, standard deviation; WBC, white blood cell.

**Table 4:** Peripheral blood cell counts in the OCRE-treated pwMS cohort during vaccination.

Blood cells	OCRE			*p value*
	T0 (mean ± sd)	T1 (mean ± sd)	T2 (mean ± sd)	
WBC (cells/µL)	6997 (± 1668)	6249 (± 1969)	6874 (± 1456)	-
Lymphocytes (cells/µL)	1657 (± 338)	1781 (± 505)	1648.9 (± 299)	-
CD20^+^ (cells/µL)	15.03 (± 30.7)	48.09 (± 82.7)	7.9 (± 20)	*,#
CD3^+^ (cells/µL)	1378 (± 329)	1459 (± 361)	1311.7 (± 283)	-
CD4^+^ (cells/µL)	853 (± 256)	927 (± 298)	818 (± 213.9)	-
CD8^+^ (cells/µL)	249 (± 48.3)	248 (± 87.5)	260.5 (± 104.5)	-
IgG Spike	0.50 (± 0.24)	27 (± 75.6)	282.5 (± 537.2)	–

The absolute number of white blood cells (WBC), lymphocytes, CD20^+^, CD3^+^, CD4^+^, and CD8^+^ in OCRE-treated MS subjects undergoing BNT162b2 vaccination was evaluated before the vaccine (T0), 21 days after the first (T1) and the second (T2) vaccine dose.

Data are presented as mean ± sd and statistical analysis was performed using the Wilcoxon signed-rank test (two tails); *;^#^;^¥^ p<0.05. * T0 *vs* T1; ^#^ T1 *vs* T2; ^¥^ T0 *vs* T2.

OCRE, Ocrelizumab; SD, standard deviation; WBC, white blood cell.

**Figure 2:**
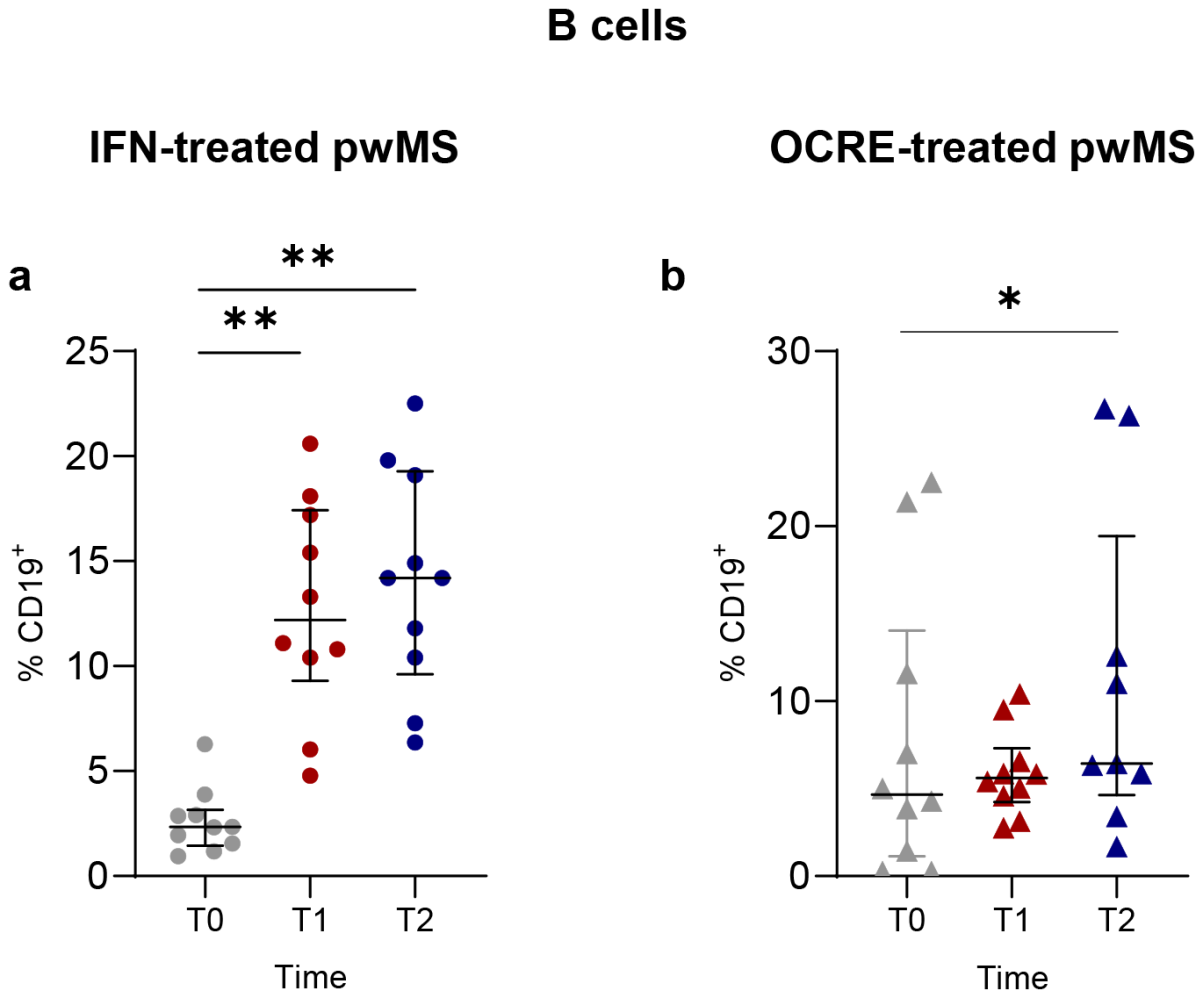
B cell evaluation in IFN- and OCRE-treated patients with multiple sclerosis (pwMS) undergoing BNT162b2 mRNA SARS-CoV-2 vaccination. Cumulative data of flow cytometry analysis showing B lymphocyte percentage, evaluated as CD19^+^ cells, in peripheral blood mononuclear cells (PBMCs) of **(a)** IFN- (●) and **(b)** OCRE-(▲) treated pwMS before (T0), 21 days after the first (T1) and the second (T2) vaccine dose. **(a)** IFN: T0: n=10; T1: n=10; T2: n= 10; **(b)** OCRE: T0: n=10; T1: n=10; T2: n= 9. Each data point represents a distinct individual (i.e., an independent biological sample). Data are presented as median values. Statistical analysis was performed by using Wilcoxon signed-rank test (two tails); *p ≤ 0.05; **p ≤ 0.01.

To better characterize the immune responses, we performed flow cytometric analysis on PBMCs to evaluate the frequency of T and B cells from IFN- or OCRE-treated pwMS after vaccination. We also examined several activation/regulatory/memory markers in the two groups of pwMS. B cell frequency (identified as CD19^+^) increased significantly at T1 and T2 in IFN- and at T2 in OCRE-treated pwMS compared to T0 (**Figures 2a, b**). Interestingly, these differences were not reflected by the CD20 cell number in the whole blood (**Table 2**), probably because the CD20 expression is more restricted than that of the B-cell-specific marker CD19. Indeed, CD20 appears later in B-cell development, at the pre-B-cell stage, and is present on mature B-cell but absent on plasmablasts and plasma cells (41–45).

**Figure 3:**
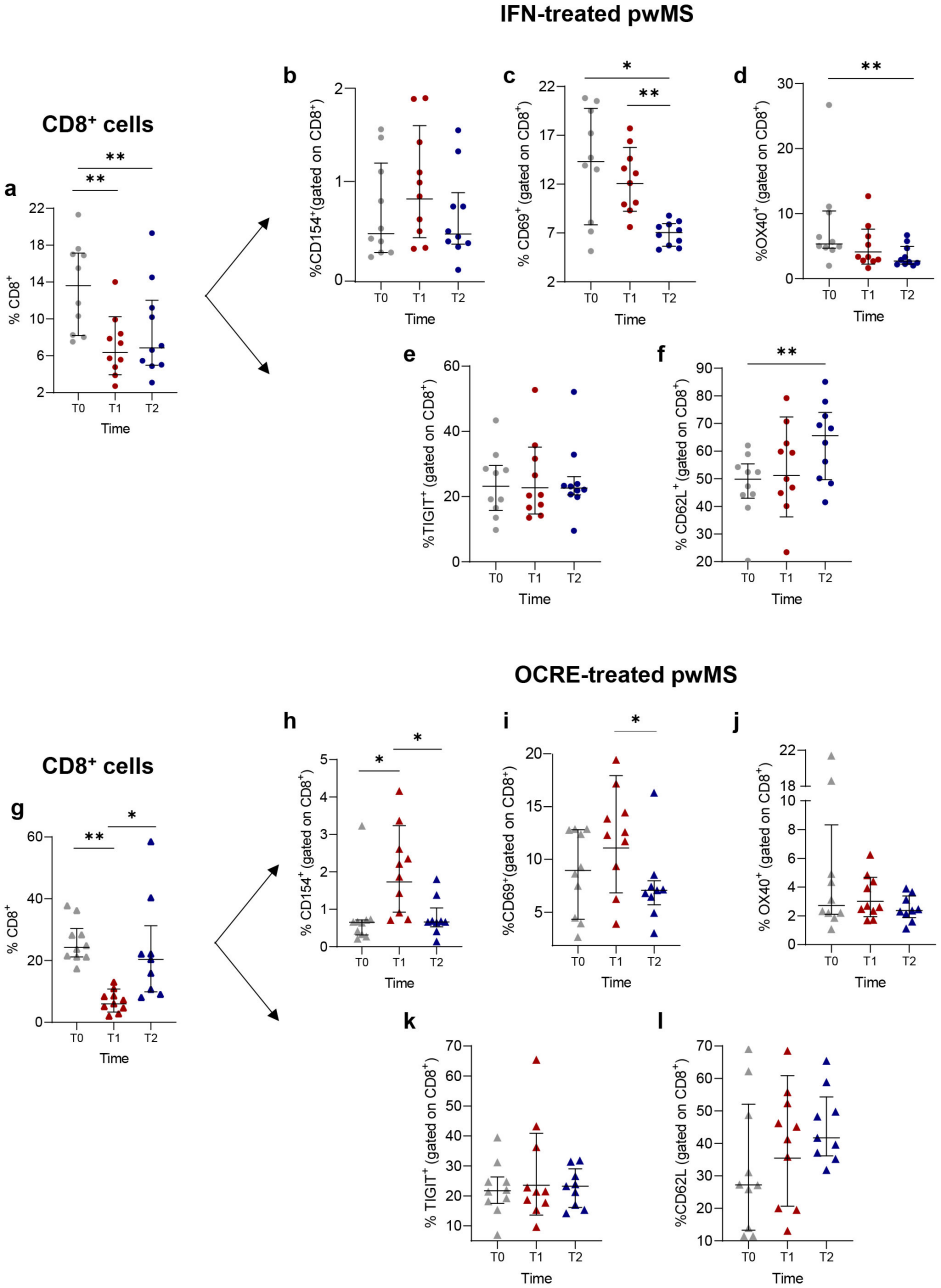
Characterization of CD8^+^ cells in IFN- and OCRE-treated patients with multiple sclerosis (pwMS) undergoing BNT162b2 mRNA SARS-CoV-2 vaccination. Cumulative data of flow cytometry analysis showing cell percentage of **(a, g)** CD8^+^, **(b, h)** CD154^+^, **(c, i)** CD69^+^, **(d, j)** OX40^+^, **(e, k)** TIGIT^+^, **(f, l)** CD62L^+^ in peripheral blood mononuclear cells (PBMCs) of **(a-f)** IFN- and **(g-l)** OCRE-treated pwMS before (T0), 21 days after the first (T1) and the second (T2) vaccine dose. **(a-f)** IFN: T0: n=10; T1: n=10; T2: n= 10; **(g-l)** OCRE: T0: n=10; T1: n=10; T2: n= 9. Each data point represents a distinct individual (i.e., an independent biological sample). Data are presented as median values. Statistical analysis was performed by using the Wilcoxon signed-rank test (two tails); *p ≤ 0.05; **p ≤ 0.01.

We also dissected the T cell compartment and found that IFN-treated pwMS showed a ~ 2-fold reduction of CD8^+^ T cell percentage both at T1 and T2 compared to baseline (T0) (**Figure 3a**). This was accompanied by a significant decrease of the CD69^+^ cell frequency at T2 compared to T1 and T0 (**Figure 3c**), and the OX40^+^ cell subset at T2 compared to T0 (**Figure 3d**). CD69 and OX40 represent early activation markers which promote the development of memory T cells (46, 47). Among all those analyzed, the frequency of CD62L^+^ cells, representing the central memory compartment (48), increased after the second dose of vaccine respect to baseline (T2 *vs* T0) (**Figure 3f**) The percentage of CD154^+^ and TIGIT^+^ cells did not change throughout the vaccination procedure (**Figures 3b, e**). TIGIT represents an immune checkpoint that downregulates hyperimmune activation (49).

OCRE-treated pwMS displayed a 3.7-fold reduction of CD8^+^ cell frequency at T1 compared to the T0 while a 3.1-fold increase at T2 *vs*. T1 (**Figure 3g**). After the first dose (T1), we found a significant increase of CD154^+^ antigen-specific activated T cell frequency (50), that was not sustained after the second dose of vaccine and a reduction of CD69^+^ cells at T2 vs T1 (**Figures 3h, i**). The percentage of OX40^+^ cells did not change during the vaccination procedure in the OCRE-treated pwMS (**Figure 3j**). Similarly, the percentage of TIGIT^+^ CD8^+^ cells was similar in T1 and T2 compared to T0 (**Figure 3k**). Finally, there was a trend of increasing CD62L^+^ cells at T1 and T2 compared to T0 although this increased frequency was not statistically significant (**Figure 3l**).

**Figure 4:**
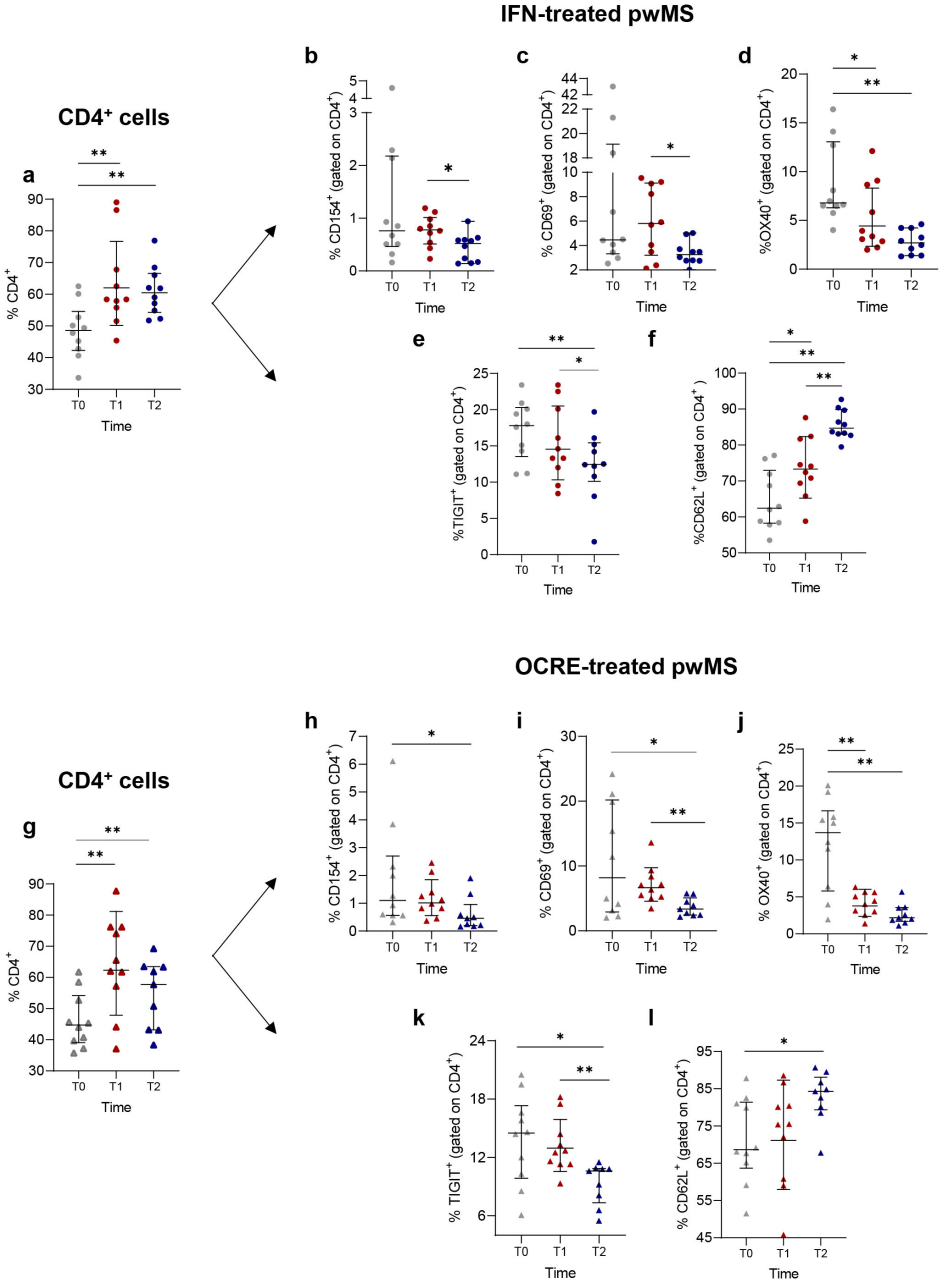
Evaluation of CD4^+^ cells in IFN- and OCRE-treated patients with multiple sclerosis (pwMS) undergoing BNT162b2 mRNA SARS-CoV-2 vaccination. Percentage of **(a, g)** CD4^+^, **(b, h)** CD154^+^, **(c, i)** CD69^+^, **(d, j)** OX40^+^, **(e, k)** TIGIT^+^, **(f, l)** CD62L^+^ cells in peripheral blood mononuclear cells (PBMCs) of **(a, f)** IFN- and **(g, l)** OCRE-treated pwMS before (T0), 21 days after the first (T1) and the second (T2) vaccine dose. **(a-f)** IFN: T0: n=10; T1: n=10; T2: n= 10; **(g-l)** OCRE: T0: n=10; T1: n=10; T2: n= 9. Each data point represents a distinct individual (i.e., an independent biological sample). Data are presented as median values. Statistical analysis was performed by using the Wilcoxon signed-rank test (two tails); *p ≤ 0.05; **p ≤ 0.01.

Then we analyzed the frequency of CD4^+^ T cell subsets in the two groups of patients. We detected a significant increase of CD4^+^ cells after both vaccine doses compared to baseline (**Figures 4a, g**). To gain more insight, we further characterized the CD4^+^ cell subsets in both pwMS groups. We found a significant reduction in the frequency of CD154^+^ and CD69^+^ cells at T2 *vs* T1 (**Figures 4b, c**), of OX40^+^ cells at T1 and T2 compared to T0, and of TIGIT^+^ cells at T2 compared to baseline and T1 in IFN-treated pwMS (**Figures 4d, e**). We also found an increased percentage of CD62L^+^ cells after the two doses of vaccine in the IFN-treated pwMS (**Figure 4f**). The latter findings support the establishment of the antigen-specific immunological memory. In the OCRE-treated group, we found a significant reduction in the frequency of CD154^+^ cells at T2 *vs.* T0 (**Figure 4h**) and a decrease of CD69^+^ cells at T2 *vs.* T1 and T0 (**Figure 4i**). OX40^+^ cells were significantly decreased after both vaccine doses compared to the baseline (**Figure 4j**). Also, TIGIT, was reduced at T2 compared to both T1 and T0 (**Figure 4k**). Finally, there was a significant increase in the frequency of CD62L^+^ cells at the T2 in OCRE-treated pwMS (**Figure 4l**).

**Figure 5:**
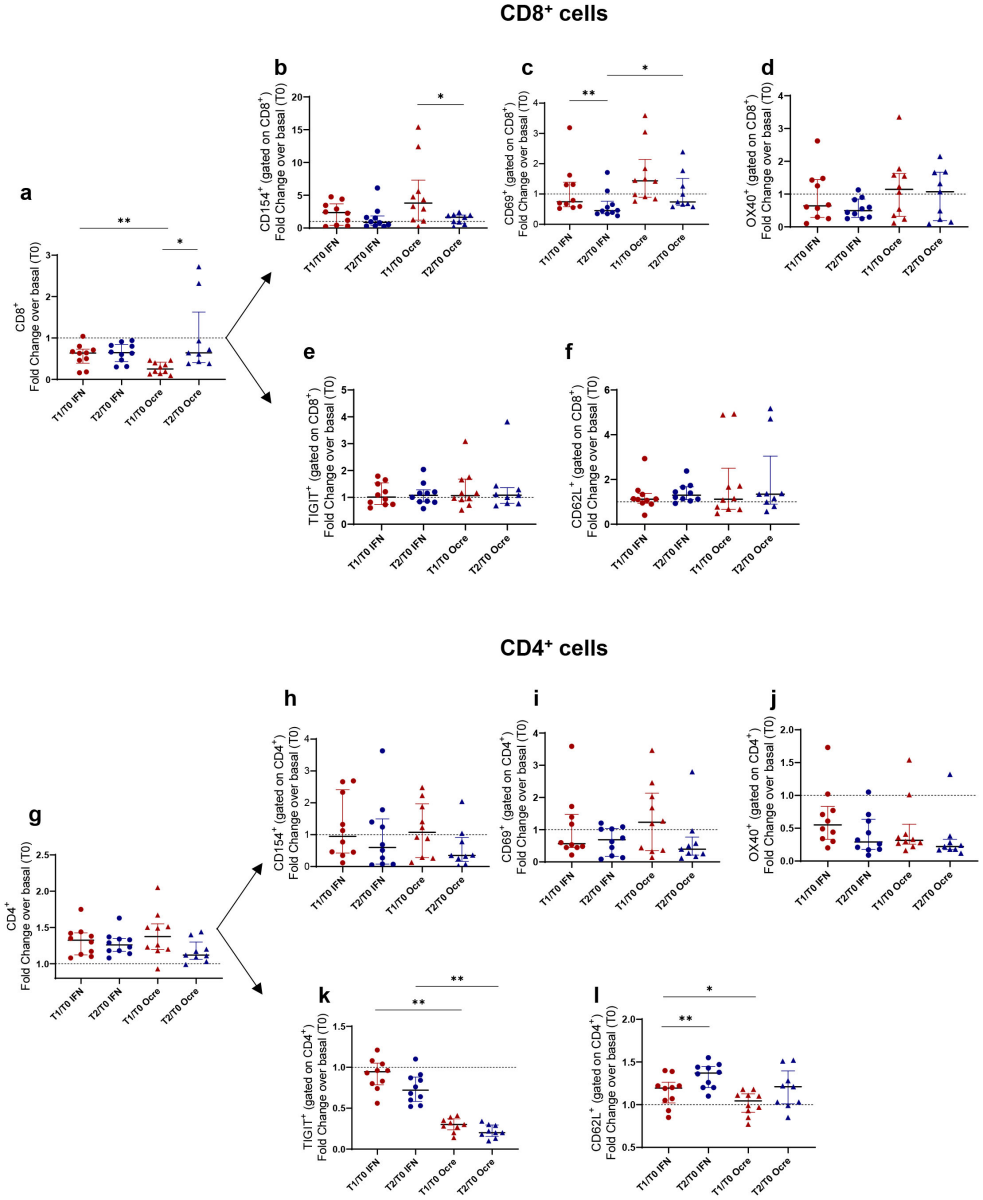
T cell comparison in IFN- and OCRE-treated patients with multiple sclerosis (pwMS) undergoing BNT162b2 mRNA SARS-CoV-2 vaccination. Fold change over basal [calculated as the ratio between the percentage of cells at T1 and T2 compared to baseline (T0)] of **(a)** CD8^+^, **(b)** CD154^+^, **(c)** CD69^+^, **(d)** OX40^+^, **(e)** TIGIT^+^, **(f)** CD62L^+^ and **(g)** CD4^+^, **(h)** CD154^+^, **(i)** CD69^+^, **(j)** OX40^+^, **(k)** TIGIT^+^, **(l)** CD62L^+^ cells in IFN (●) and OCRE (▲) -treated pwMS. Data are presented as median values. Statistical analysis was performed by using the Kruskal-Wallis test. *p ≤0.05, **p ≤0.01.

Next, we wondered whether the two DMTs could differentially impact the protective SARS-CoV-2 vaccine response over time, evaluating the *ratio* between the percentage of cells at T1 and T2 compared to baseline (T0) in two groups of patients. Compared to IFN, we found that vaccination led to a significant decrease of CD8^+^ cells in the OCRE-treated group (**Figure 5a**). Further analysis also revealed a reduction of the TIGIT^+^ and CD62L^+^ in the CD4^+^ T cells of the OCRE-treated pwMS (**Figures 5k, l**). The other differences were detected only within the same treated group of pwMS (**Figures 5b, c, l**). These data pointed out the different peripheral immune profiles shaped by the specific DMT therapy in pwMS undergoing the BNT162b2 vaccine.

### 3.3 T helper (Th)-17 and T regulatory cells (Tregs) in OCRE- and INF-treated pwMS undergoing BNT162b2 vaccination

**Figure 6:**
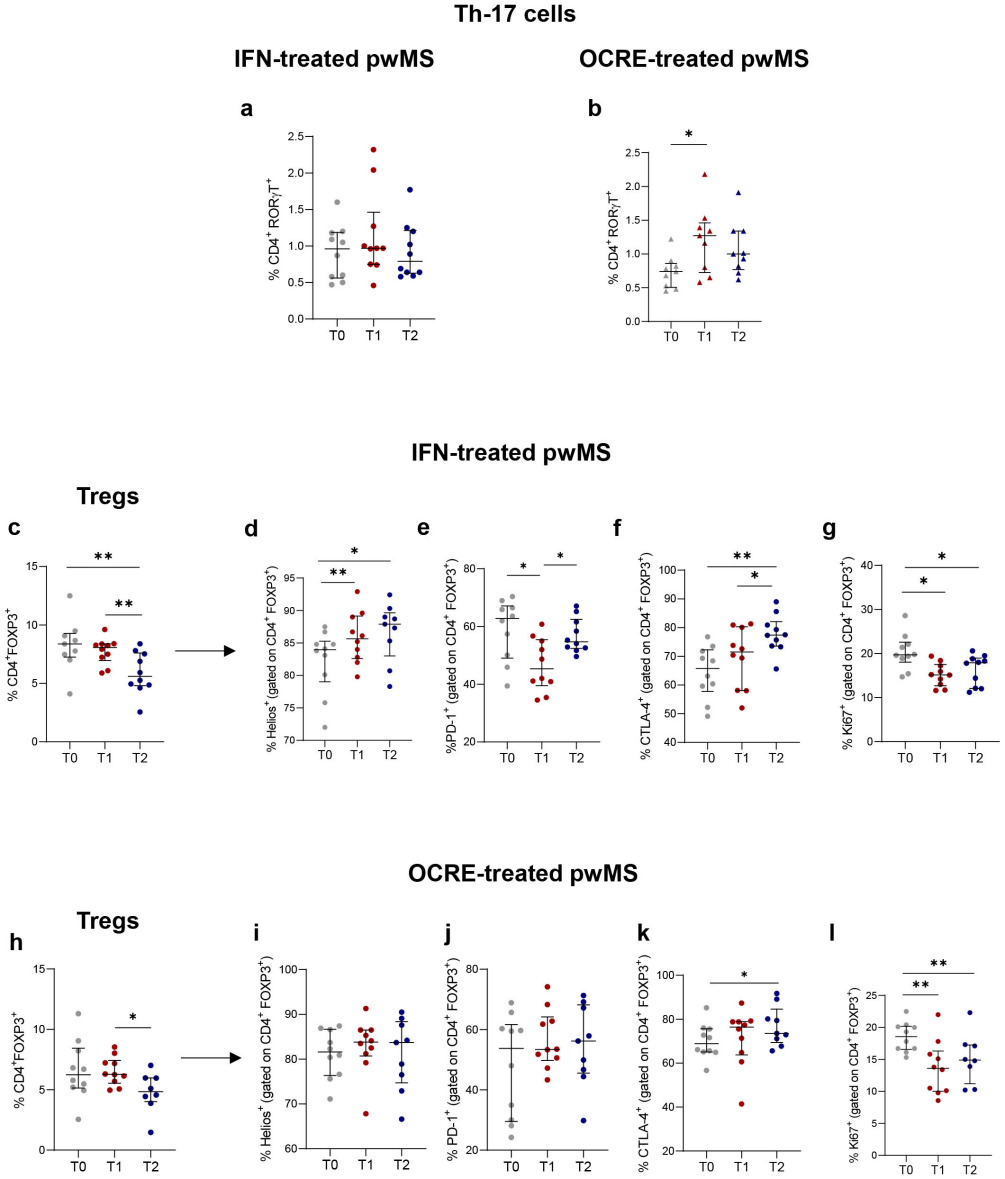
Th17 cell percentage and characterization of T regulatory cells (Tregs) in IFN- and OCRE-treated patients with multiple sclerosis (pwMS) undergoing BNT162b2 mRNA SARS-CoV-2 vaccination. Cumulative data of flow cytometry analysis showing **(a, b)** the percentage of Th17 cells (gated on CD4^+^ and identified as CD4^+^RORɣT^+^) and **(c, h)** Treg cells (gated on CD4^+^ and identified as CD4^+^FOXP3^+^), as well as the percentage of **(d, i)** Helios^+^, **(e, j)** PD1^+^, **(f, k)** CTLA-4^+^ and **(g, l)** Ki67^+^ cells in peripheral blood mononuclear cells (PBMCs) of **(a, c–g)** IFN- and **(b, h–l)** OCRE-treated pwMS before (T0), 21 days after the first (T1) and the second (T2) vaccine dose. **(a, c–g)** IFN: T0: n=10; T1: n=10; T2: n=10; **(b, h–l)** OCRE: T0: n=10; T1: n=10; T2: n=9. Each data point represents a distinct individual (i.e., an independent biological sample). Data are presented as median values (for panels **(c–l)**, median values of at least n=8 subjects). Statistical analysis was performed by using the Wilcoxon signed-rank test; *p ≤0.05, **p ≤0.01.

We also characterized two CD4^+^ T cell subsets, Th17 (identified as RORγT^+^ cells) and Tregs (identified as CD4^+^FOXP3^+^), that play a key role in MS pathogenesis and protection (51–54). FACS analysis revealed a significant increase in Th17 cell frequency after the first dose in the OCRE-treated pwMS group. By contrast, the percentage of Th17 cells remained unchanged during vaccination (T1 and T2) compared to baseline (T0) in the INF-treated pwMS (**Figures 6a, b**).

We also characterized the Treg subset in the two groups of patients. We found a comparable reduction in Treg frequency in both groups of pwMS after the second vaccine dose (T2) compared to T1 (**Figures 6c, h**). These data are in line with a recent study suggesting that Treg reduction is associated with improved vaccination efficacy in pwMS (9, 55). We also performed a more detailed characterization of the Treg subsets. The decreased frequency of Tregs was accompanied by higher expression of Helios after both vaccine doses compared to the baseline and of CTLA-4 at T2 *vs.* T1 and T0 in IFN-treated pwMS (**Figures 6d, f**). In this group of pwMS, PD-1^+^ cell frequency diminished at T1 compared to T0; however, the expression of PD-1^+^ was restored after the second vaccine dose (**Figure 6e**). Moreover, Tregs derived from the IFN-treated group were less proliferative as testified by the reduction of Ki67 expression at both T1 and T2 compared to T0 (**Figure 6g**). Helios^+^ and PD-1^+^ cell frequency did not change in the OCRE-treated pwMS (**Figures 6i, j**). In this group of patients, CTLA^+^ cells increased after the second vaccine dose (T2) compared to baseline (T0) (**Figure 6k**). Similarly to the IFN-treated patients, there was a significant reduction of Ki67^+^ cell frequency after both vaccine doses (T1 and T2) in OCRE-treated pwMS (**Figure 6l**).

Collectively, these findings pointed out that SARS-CoV-2 vaccination differentially impacts T cell subsets in these two pwMS cohorts, determining an increase of Th17 and a reduction of Tregs.

### 3.4 Spike-specific immune response in CD8^+^ and CD4^+^ T cells from OCRE- and IFN-treated pwMS undergoing BNT162b2 vaccination

To measure the antigen (spike)-specific immune response in CD8^+^ and CD4^+^ T cells, we evaluated the expression of activation-induced marker (AIM) and intracellular cytokine secretion (ICS) in PBMCs isolated from pwMS and stimulated with a pool of peptides spanning the entire spike protein (56). Spike reactivity was evaluated as co-expression of CD69 and CD137 on CD8^+^ cells, and co-expression of CD154 and OX40 on CD4^+^ cells; we also measured IFN-γ, TNF-α, and IL-2 production, the dominant cytokines in spike-specific T cells (57).

**Figure 7:**
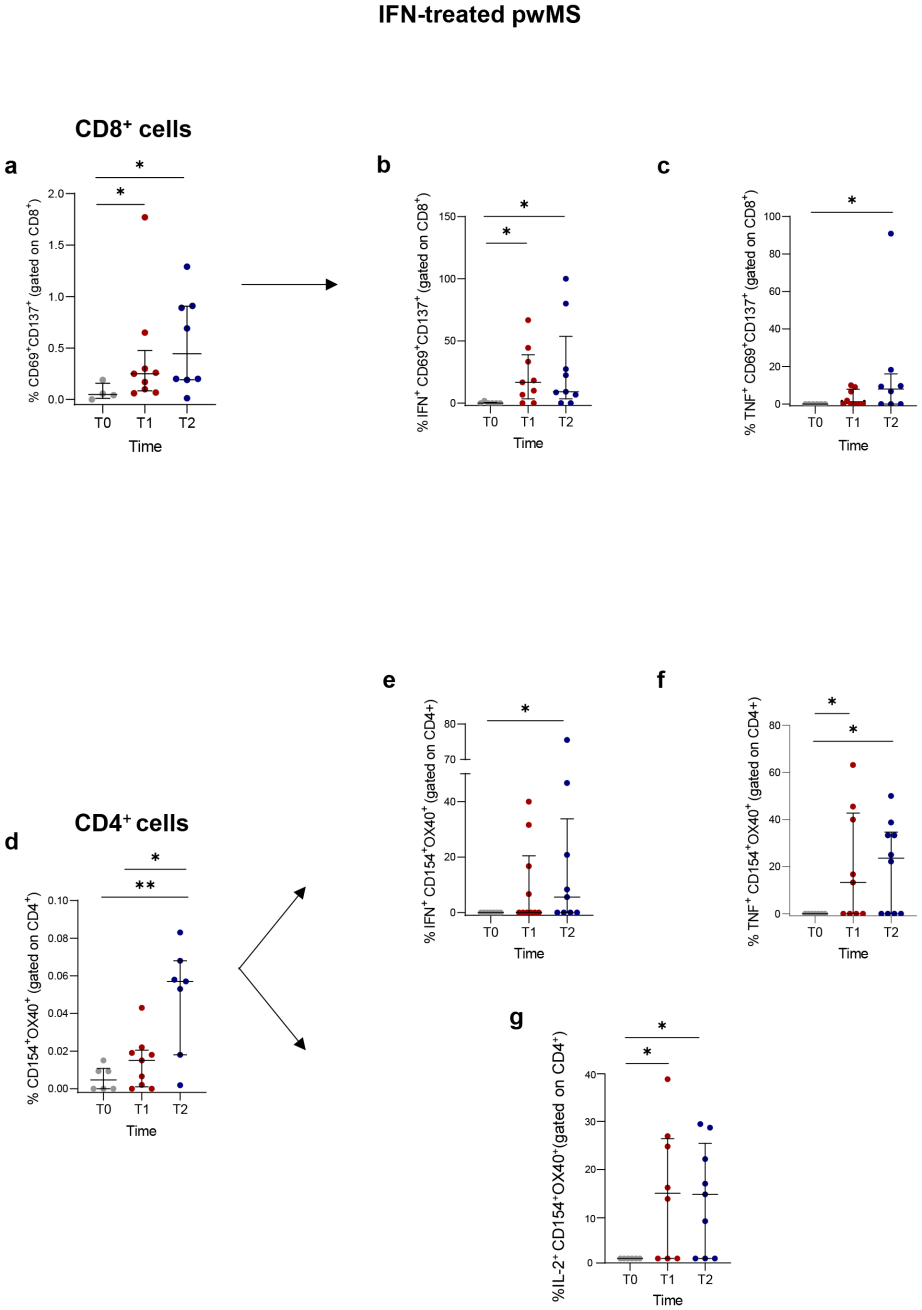
CD8^+^ and CD4^+^ T cell response in INF-treated patients with multiple sclerosis (pwMS) before and after BNT162b2 mRNA SARS-CoV-2 vaccination. Cumulative data of flow cytometry analysis of spike-specific CD8^+^ and CD4^+^ T cells measured as the percentage of activated **(a)** CD69^+^CD137^+^ and **(d)** CD154^+^OX40^+^ T cells, respectively. Intracellular cytokines evaluation of **(b)** INF-γ and **(c)** TNF-α in CD8^+^ antigen-specific T cells and **(e)** IFN-γ, **(f)** TNF-α, and **(g)** IL-2 in CD4^+^ antigen-specific T cells. Analysis was performed after stimulation for 18 hours at 37 °C with a peptide pool spanning the entire Spike sequence. Data are presented as median values of at least n=4 subjects. Statistical analysis was performed by using the Mann-Whitney *U*-test (two tails); *p ≤0.05, **p ≤0.01.

**Figure 8:**
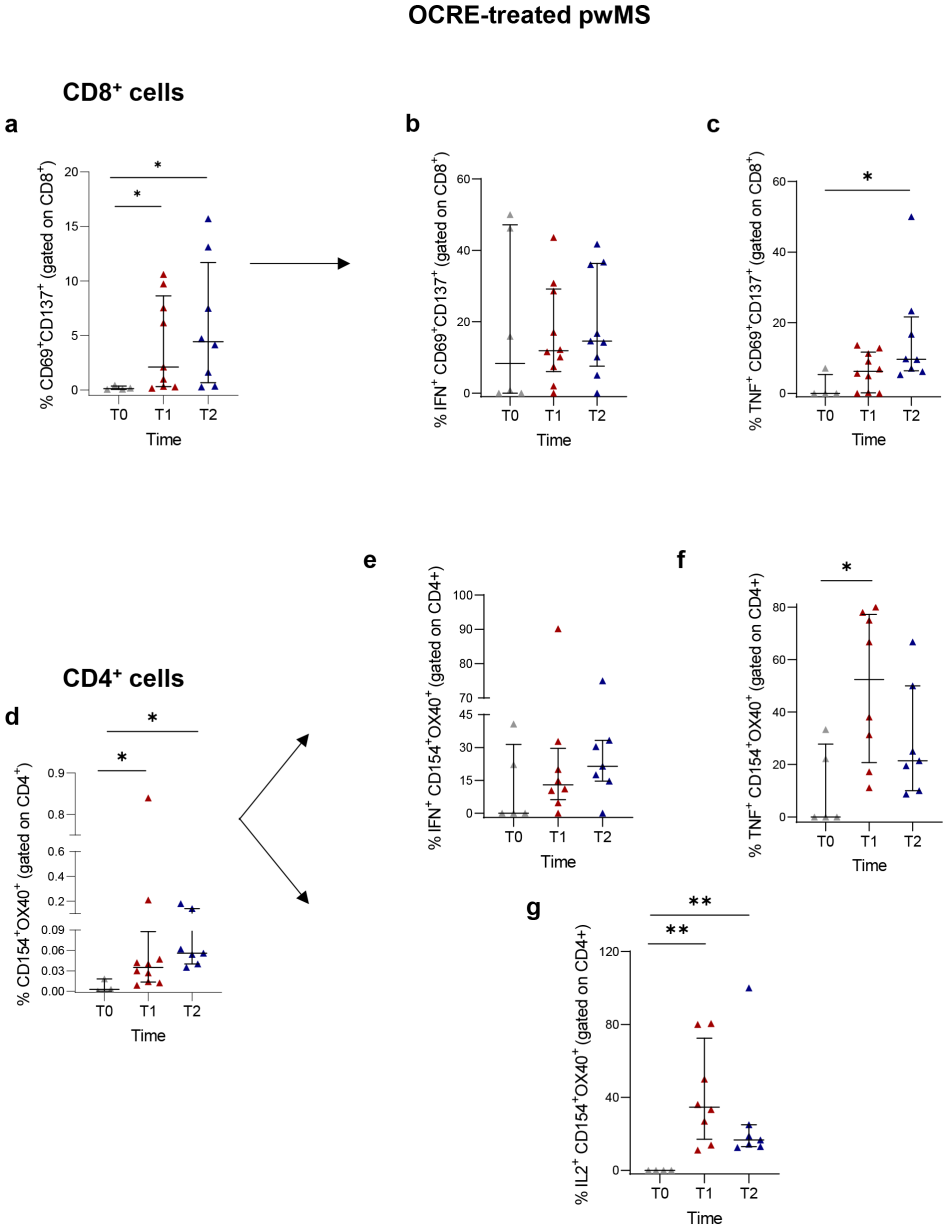
CD8^+^ and CD4^+^ T cell response in OCRE-treated patients with multiple sclerosis (pwMS) before and after BNT162b2 mRNA SARS-CoV-2 vaccination. Cumulative data of flow cytometry analysis of spike-specific CD8^+^ and CD4^+^ T cells measured as the percentage of activated **(a)** CD69^+^CD137^+^ and **(d)** CD154^+^OX40^+^ T cells, respectively. Intracellular cytokines evaluation of **(b)** IFN-γ and **(c)** TNF-α in CD8^+^ antigen-specific T cells and **(e)** IFN-γ, **(f)** TNF-α, and **(g)** IL-2 in CD4^+^ antigen-specific T cells. Analysis was performed after stimulation for 18 hours at 37 °C with a peptide pool spanning the entire Spike sequence. Data are presented as median values of at least n=3 subjects. Statistical analysis was performed by using the Mann-Whitney *U*-test (two tails); *p ≤0.05, **p ≤0.01.

In IFN-treated pwMS, the percentage of CD69^+^CD137^+^ cytotoxic cells significantly increased at T2 and T1 compared to T0 (**Figure 7a**). Further analysis also revealed a significant production of IFN-γ at T2 and T1 and of TNF-α at T2 compared to the baseline (**Figures 7b, c**). Moreover, the percentage of CD154^+^OX40^+^ in CD4^+^ cells and cytokine production were significantly higher at T2 compared to both T1 and T0 (**Figure 8d**). In particular, IFN-γ production increased at T2 (**Figure 7e**), while TNF-α and IL-2 increased at both T2 and T1 compared to T0 (**Figures 7f, g**).

OCRE-treated pwMS showed a 17-fold increase in the frequency of double-positive CD69^+^CD137^+^ cytotoxic cells at T1 and a 36-fold increase at T2 compared to the baseline (**Figure 8a**), paralleled by a marked TNF-α production at T2 (**Figure 8c**). IFN-γ production did not change throughout the vaccination (**Figure 8b**). There was also a significant increase of CD154^+^OX40^+^ cell frequency after both vaccine doses compared to the baseline (**Figure 8d**). Double-positive CD154^+^OX40^+^ producing INF-γ did not change throughout the vaccination (**Figure 8e**). Cytokine analysis revealed an early increase of TNF-α and IL-2 at T1 compared to T0 by spike-specific CD4^+^ cells (**Figures 8f, g**).

Overall, our results revealed that pwMS treated with IFN and OCRE mount an adequate SARS-CoV-2-specific immune response after vaccination, both in terms of effector T cell function and immunological memory.

## 4 Discussion

Vaccine-induced humoral and cellular responses are mandatory for viral clearance and a coordinated and efficient immune activity. In particular, SARS-CoV-2 vaccination and the elicited immune responses are of paramount importance for pwMS who exhibit a variable vaccine-induced response influenced by DMTs (20, 38, 58). Several studies evaluated the effectiveness of anti-COVID-19 vaccination in DMT-treated pwMS, showing that most, but not all of these therapies, allows to mount a protective immune response (13–19, 22, 25). Importantly, the SARS-CoV-2 virus frequently undergoes mutations, giving rise to novel strains, which can escape immunity. Therefore, the characterization of anti-SARS-CoV-2 vaccine immune response in pwMS treated with DMT is essential not only to understand its effects and efficacy in a vulnerable population, but also to act promptly in the event of emergency of new variants or second pandemic.

To dive into these aspects, in this longitudinal monocentric study, we made a broad characterization of the immune phenotype and the antigen (spike)-specific T cell responses in IFN- and OCRE-treated pwMS undergoing BNT162b2 vaccination. We compared the vaccine-immune response in two groups of pwMS treated with IFN or OCRE, which display different mechanism of action. Previous studies have shown that the vaccine-induced humoral response in IFN-treated pwMS is comparable to normal donors (20, 25, 37, 39), while in OCRE-treated patients is still controversial (13–16, 22–24). Notwithstanding, no increased risk of SARS-CoV-2 infection has been described in OCRE-treated pwMS (59, 60).

Our findings revealed a reduction of B cell number in the OCRE-treated group, according to its mechanism of action, depleting circulating B-cells (61). We also confirmed the reduced humoral response to SARS-CoV-2 mRNA vaccination, previously reported in OCRE-treated pwMS (23, 38–40). Our detailed analysis of the T cell compartment revealed a reduction of CD8^+^ cells in both pwMS groups, accompanied by a decrease of activation markers (i.e., CD69 and OX40) in IFN-treated group and no changes in OCRE-treated pwMS. Interestingly, we found a transient increase of CD154^+^ cells selectively in the OCRE-treated pwMS following the first vaccine dose. CD154 is a member of the TNF superfamily of molecules playing an important role in CD8^+^ T cell responses, mainly in the generation of memory CD8^+^ T cells (62). This observation could suggest an augmented immunological memory in OCRE-treated pwMS, to counterbalance their humoral impairment. The progressive increased percentage of CD62L^+^ cells in the IFN-treated group is a hallmark of central memory T cells, which have long-term persistence and can be quickly mobilized to respond to a second antigen exposure (63). This observation further reveals the capacity of the immune system to mount a proper vaccine-elicited immune response with the generation of memory T cells in IFN-treated pwMS.

When we examined the CD4^+^ compartments, we observed their increase after both vaccine doses in the IFN- and after the first dose in the OCRE-treated group. Interestingly, the expression of all activation markers examined (CD154, CD69, OX40) was progressively reduced after vaccination in both groups of pwMS. By contrast, vaccination induced a continuous increase in the frequency of memory CD4^+^ lymphocytes, detected as CD62L^+^ cells in both pwMS groups. Analysis of the CD4^+^ cell compartment revealed a significant decrease in the frequency of TIGIT^+^ cells in OCRE- *vs* IFN- treated pwMS. TIGIT is an immune checkpoint protein able to suppress T-cell activation, hindering the body’s defence mechanisms against infections (64). Additionally, the TIGIT axis triggers immune tolerance by inhibiting autoreactive T cells, fostering the development of tolerogenic dendritic cells, and enhancing the suppressive capacity of Tregs (65). Checkpoint inhibitors have been shown to enhance the anti-viral immune response and to boost the virus-specific T cell response (66). In this complex scenario, the downregulation of TIGIT in OCRE-treated pwMS could unveil a specific mechanism to boost T cell responses to counterbalance the B cell defect. The comparison between the two groups also uncovered a reduction of CD4^+^CD62L^+^ cells in the OCRE- *vs* IFN- treated pwMS at T1, suggesting a delayed induction of this memory subset in the OCRE-treated group. Collectively, these results highlight that, upon vaccination, IFN- and OCRE-treated pwMS mounted a consistent immune response with the establishment of immunological memory. By comparing IFN- *vs* OCRE-treated pwMS after the first vaccine dose, we detected a reduction of CD8^+^ cells in the OCRE-treated group, associated with an increase in memory CD8^+^ frequency (i.e., CD154^+^); this highlights the activation of the adaptive immune system in response to vaccination.

The Th17 cells’ involvement in the vaccine-elicited immune response is of utmost importance for pwMS, given their key role as the subset accounting for disease pathogenesis (51, 67, 68). Increasing evidence points to a critical function for Th17 inflammatory responses also in the pathogenesis of COVID-19 (69, 70). In particular, several studies have investigated the effects of SARS-CoV-2 vaccination on the Th17 subset, revealing high levels of Th17 cells in the peripheral blood of individuals SARS-CoV-2 positive (70–72). While the inhibitory effect of IFN on human Th17 differentiation has been reported (73–75), the impact of OCRE on Th17 compartment in pwMS is still unknown. Our results show for the first time that vaccination induced an increase of Th17 cells in OCRE-treated pwMS, while this subset remained stable in the IFN-treated group throughout vaccination. Further studies should evaluate whether the increased frequency of Th17 cells in OCRE-treated pwMS is transient and associated with functional activity.

The number and function of Tregs are impaired in pwMS, which may contribute to the development and progression of the disease (76–79). Moreover, a reduction of Treg frequency has been reported in patients with severe COVID-19 (80). It has been suggested that Tregs could interfere with the generation of vaccine-induced immunity (81). Although IFN-β promotes the proliferation of Tregs, how these cells impact the vaccine immunogenicity in pwMS remains to be fully determined (82). Tregs are induced by infections to regulate the inflammatory response and could be induced as part of the immune response to vaccination (55, 83). Moreover, a reduction of Treg frequency is associated with improved vaccination efficacy in pwMS (8, 9, 55). To the best of our knowledge, our study revealed for the first time a reduction of Tregs in both pwMS groups following SARS-CoV-2 vaccination. The decrease of Tregs was accompanied by a more stable and suppressive phenotype only in the IFN-treated group, as indicated by the increased expression of Helios, PD-1, and CTLA-4; this could represent a compensatory mechanism to face the reduction of Tregs and control the vaccine-induced immune response. Interestingly, these compensatory mechanism was limited to CTLA-4 in the OCRE-treated group.

We also evaluated the spike-specific immune response in CD8^+^ and CD4^+^ T cells from both groups of pwMS. Interestingly, we found that the antigen-specific vaccine response was preserved in both groups. Spike-specific cytotoxic CD8^+^ cells (CD69^+^CD137^+^ double-positive cells) increased more markedly in the OCRE-treated group compared to IFN. This observation is consistent with prior findings revealing an increase of SARS-CoV-2-specific CD8^+^ T cell activation in OCRE-treated pwMS (56), suggesting a compensatory mechanism to overcome the B cell defect. In addition, spike-specific CD8^+^ and CD4^+^ T cells released effector cytokines (i.e., IFN, TNF-α), further supporting that IFN and OCRE treatments do not alter T cell polyfunctionality.

We would like to mention that this study has some limitations that should be pointed out. First, the small sample size of pwMS examined, and the lack of comparison with other DMTs reflect, at least in part, the complexity of the deep immunological phenotyping and functional characterization to assess the SARS-CoV-2 response. Another critical aspect could be the longer treatment duration of the IFN-treated pwMS group and the different male/female *ratio* in the two pwMS groups. The first limitation could be explained since IFN is a first-line drug, and stable MS patients are treated with this drug for long time. The increased female proportion in the IFN group could be explained by the evidence that this treatment is preferred before/during pregnancy and breastfeeding, due to its more favourable safety profile (84–86). A final aspect that should be mentioned is the different administration schedule of OCRE and IFN in pwMS. In this regard, we followed the recommendations of the Italian Authority of Health, providing tailored indications to optimize vaccine immunogenicity while ensuring treatment continuity (33). Overall, the demographic and clinical characteristics differ substantially between the two groups and this may affect vaccine-induced immune responses.

In conclusion, our study offers significant insights by illustrating a broad immunophenotyping and the functional response to the SARS-CoV-2 vaccine in pwMS undergoing two most common DMTs, monitored throughout vaccination. We have demonstrated in detail that although IFN and OCRE induce distinct immunological profiles, both DMTs allowed to mount a proper vaccine-induced cellular immune response. Our results support the evidence that MS patients undergoing IFN or OCRE treatments are immunocompetent, and consequently, SARS-CoV-2 vaccination is crucial for protecting these at-risk individuals from severe infection outcomes. The information emerging from this study will also be of importance in the event of emergency of evolving dominant SARS-CoV-2 variants. Moreover, this study provides an immunological characterization of the spike-specific T-cell response in pwMS under IFN and OCRE, evaluating immunological biomarkers whose relevance may extend beyond COVID-19 for studying immune responses to other infections and vaccinations. Finally, in a broader view, the methodological approach and the results of this study could also be useful to guide the evaluation of the immunomodulatory effects of biologics increasingly used in several immune disorders.

## Data Availability

The raw data supporting the conclusions of this article will be made available by the authors, without undue reservation.
